# Exploring the Associations Between Dysphagia and Health-Related Outcomes in Older Adults: Results from the ilSirente Study

**DOI:** 10.3390/nu17132149

**Published:** 2025-06-28

**Authors:** Hélio José Coelho-Júnior, Alejandro Álvarez-Bustos, Cristina Pérez Ramírez, Andrea Russo, Leocadio Rodriguez-Mañas, Francesco Landi, Emanuele Marzetti

**Affiliations:** 1 Fondazione Policlinico Universitario Agostino Gemelli IRCCS, 00168 Rome, Italy; andrea.russo1@policlinicogemelli.it (A.R.); francesco.landi@unicatt.it (F.L.); 2 Department of Geriatrics, Orthopedics and Rheumatology, Università Cattolica del Sacro Cuore, L.go F. Vito 1, 00168 Rome, Italy; 3 Biomedical Research Center Network for Frailty and Healthy Ageing (CIBERFES), Institute of Health Carlos III, 28029 Madrid, Spain; a.alvarezbu@gmail.com (A.Á.-B.); leocadio.rodriguez@salud.madrid.org (L.R.-M.); 4 Department of Geriatrics, Hospital Universitario de Getafe, 28905 Madrid, Spain; perezcristina1510@gmail.com; 5 Instituto de Investigación IdiPaz, 28029 Madrid, Spain

**Keywords:** frailty, sarcopenia, multimorbidity, polypharmacy, nutritional status, cognitive function

## Abstract

**Objectives:** The present study examined cross-sectional and longitudinal associations between dysphagia and a variety of health-related parameters, including physical performance, cognitive function, malnutrition, sarcopenia, disability, frailty, falls, hospitalization, and mortality in a cohort of octogenarians living in the mountainous Sirente region of Central Italy. **Methods:** Dysphagia was operationalized as the need to modify the diet to facilitate swallowing and/or the exclusive consumption of specific food consistencies due to swallowing difficulties. Physical performance, cognitive function, malnutrition, disability, falls, and hospitalizations were assessed via the Minimum Data Set for Home Care. Sarcopenia was defined as the coexistence of low muscle mass and dynapenia, while frailty was operationalized according to Fried’s phenotype. History of falls and incident falls, as well as disability, were tracked over two years, while survival status was followed for up to ten years. **Results:** Data of 362 older adults (men age: 85.9 ± 4.8; body mass index: 25.6 ± 4.53; women: 66.9%; multimorbidity: 21.5%; dysphagia: 6.6%) were analyzed. The results indicated that dysphagia was significantly and cross-sectionally associated with poor physical performance and reduced cognitive function. In contrast, no longitudinal associations were observed. **Conclusions:** Dysphagia appears to be linked to deficits in physical and cognitive domains, underscoring the value of comprehensive geriatric assessment and the development of multidomain intervention strategies.

## 1. Introduction

Dysphagia is a clinical condition characterized by difficulty in or inability to safely and effectively swallow liquids, food, and/or medications [1]. It is a multifactorial condition, with potential causes including neurological disorders (e.g., stroke), dementia, and the use of certain medications [1]. Dysphagia is highly observed worldwide, with an estimated prevalence of 43.8% in individuals 18+ years [2]. This high prevalence persists when estimations are examined according to each continent, with notorious rates found in Africa (64.2%), America (51.3%), and Europe (45.7%) [2]. In Italy, data from the INDEED project indicate that over six million people suffer from dysphagia, affecting approximately one in five individuals [3]. Among the populations more affected by dysphagia, older adults appear particularly vulnerable due to the convergence of multiple risk factors. In community-dwelling older adults, the estimated prevalence ranges from 15% to 30.5%, depending on the assessment method used [4,5].

This scenario is concerning because the progression of dysphagia can precipitate a range of adverse outcomes. For instance, swallowing impairments often lead to insufficient caloric and protein intake, resulting in malnutrition [6] and declines in physical performance and muscle mass [6], predisposing to the occurrence of sarcopenia and frailty [7]. As dysphagia worsens, these factors further elevate the risk of falls and disability [4]. Another important concern regarding dysphagia is that it compromises swallowing safety, increasing the risk of aspiration pneumonia and, consequently, hospitalization [1]. Notably, some evidence has also suggested that the progression of dysphagia might reflect cognitive impairments, mainly in attention and inhibition domains, as the neural control of swallowing overlaps with brain networks involved in mental abilities [8,9,10].

Despite the theoretical link between dysphagia and adverse health outcomes, empirical research detailing the factors associated with this condition remains scarce. A recent systematic review identified only 11 studies on dysphagia in community-dwelling older adults [5]. Moreover, findings from studies examining the associations between dysphagia and various health parameters have been inconsistent. Indeed, some investigations have not supported significant associations between dysphagia and many adverse outcomes, such as sarcopenia [11] and cognitive impairments [12]. Furthermore, although a pooled analysis found that dysphagia was associated with a significantly increased risk of prefrailty and frailty [13], no associations were observed when frailty was analyzed alone [13]. Therefore, additional studies are needed to clarify the relationship between dysphagia and a broad range of health outcomes in older adults, particularly in real-world, community-based settings.

To contribute to this knowledge gap, the present study investigated both cross-sectional and longitudinal associations between dysphagia and several health-related parameters, including physical performance, cognitive function, malnutrition, sarcopenia, disability, frailty, falls, hospitalization, and mortality.

## 2. Materials and Methods

The current investigation utilized data from the Aging and Longevity Study in the Sirente Geographic Area (ilSIRENTE) database [14]. This prospective cohort study was carried out in the mountain community of the Sirente region, located in Central Italy (L’Aquila, Abruzzo, Italy). The population in this region resides in 13 towns and villages, situated at elevations ranging from 800 to 1400 m above sea level and surrounded by mountainous terrain. The ilSIRENTE study was developed collaboratively by the Department of Geriatrics at Università Cattolica del Sacro Cuore (Rome, Italy) and the teaching nursing home Opera Santa Maria della Pace (Fontecchio, L’Aquila, Italy), in cooperation with local administrators and primary care physicians from the Sirente Mountain Community Municipalities.

The research adhered to the ethical guidelines outlined in the Declaration of Helsinki and received approval from the Ethics Committee of Università Cattolica del Sacro Cuore (A.0834/CF/2003, date of approval: 16 October 2003). Written informed consent was obtained from all participants or their legal representatives, when necessary, prior to enrolment.

### 2.1. Participants

In October 2003, a comprehensive list of residents in the Sirente area was obtained from the registry offices of all municipalities involved in this study. Registration in these municipal records is mandatory at birth or upon relocation, as it is required to access primary health care services. This requirement ensured complete population coverage, including individuals residing in nursing homes.

From the list, potential study participants were identified by selecting those living in the Sirente area who were born before 1 January 1924. Among the 514 individuals initially screened, 32 men and 53 women had either died or moved away prior to baseline assessment. General practitioners explained the ilSIRENTE study protocol to their eligible patients and invited them to participate. Individuals who initially declined were contacted at least two additional times by the study personnel before being definitively classified as refusals.

Of the 429 eligible individuals, the refusal rate was low (16%), with no significant differences by age or sex. As a result, a total of 364 participants aged 80 years and older were enrolled in the study. For the present analysis, participants who provided data regarding dysphagia and were not using tube feeding were included (*n* = 362).

### 2.2. Data Collection

Baseline data collection began in December 2003 and concluded in September 2004. Participants underwent clinical interviews and functional assessments at specific research clinics located in each town. When participants were unable to attend in person—due to mobility limitations, cognitive issues, or transportation barriers—evaluations were conducted in their homes. Information regarding medical history, current medications, and lifestyle habits was collected using standardized and validated questionnaires. All assessments were administered by a multidisciplinary team, including physicians, nurses, physiotherapists, medical residents, and students from the Department of Geriatrics at Università Cattolica del Sacro Cuore, in collaboration with the Opera Santa Maria della Pace nursing home and local general practitioners. The ilSIRENTE study database is overseen by the principal investigator (F.L.).

### 2.3. Dysphagia

Dysphagia was assessed using item 3 of section L of the Minimum Data Set for Home Care (MDS−HC) instrument [15]. Participants or their proxies were asked to indicate whether any dietary modifications were made to facilitate the ingestion of solid foods (e.g., puree) and/or liquids (e.g., thickened liquids), or whether only specific food groups could be consumed due to the presence of swallowing problems. The diagnosis was confirmed by a health professional, either a physician or a nurse, responsible for data collection.

### 2.4. Outcomes

#### 2.4.1. Cognitive Function

Cognitive function was evaluated by summing scores from specific items in the Section B (items 1–3) of the MDS−HC [15]. These items enquire about the presence of memory problems, current capacity to make decisions independently and worsening in decision making in the past 90 days before data collection, changes in mental function in the week prior to the assessment, and presence of delirium. High total scores in this parameter indicate greater impairment.

#### 2.4.2. Malnutrition

Malnutrition was operationalized according to the presence of at least one phenotypic and at least one etiologic criterion, as recommended by the GLIM criteria [16]. Phenotypic criteria included the following parameters: (a) unintentional weight loss ≥5% in the last 30 days or ≥10% in the last 180 days; (b) low body mass index (BMI, <22 kg/m^2^); and (c) low muscle mass (appendicular skeletal muscle (ASM) < 20 kg for men and <15 kg for women). Etiologic criteria included (a) reduced food intake (answered “Yes, a little” or “Yes, a lot” to the question “The amount of food you usually eat has decreased over the last year?”); and (b) inflammation (*C*-reactive protein (CRP) values ≥9 mg/L) [17].

#### 2.4.3. Physical Performance

The short physical performance battery (SPPB) was performed under standardized conditions [18]. The battery involves three tests that evaluate lower-body function: a hierarchical test of standing balance, a 4 m walk speed (WS) test, and the five times sit-to-stand (5STS) test. Each SPPB subtest is scored from 0 to 4, with 0 representing the inability to perform the test and a score of 4 representing the highest category of performance.

#### 2.4.4. Sarcopenia

Sarcopenia was defined as the concurrent presence of dynapenia and low ASM, based on the cutoff points recommended by the European Working Group on Sarcopenia in Older People (EWGSOP) guidelines [19]. Dynapenia was assessed using both isometric handgrip strength (IHG) and the 5STS tests, according to the cutoff points proposed by the EWGSOP [19]. ASM was estimated using the formula proposed by the COCONUT Study Group, based on calf circumference measurements [20].

#### 2.4.5. Frailty

Frailty was operationalized according to a modified Fried phenotype, based on the presence of three or more of the following criteria: (i) WS below 0.8 m/s; (ii) reduced muscle strength (IHG < 30 kg for men and <20 kg for women); (iii) unintentional weight loss (5% or more in the past 30 days, or 10% or more in the past 6 months); (iv) low energy expenditure (defined as engaging in less than one hour per week in activities such as walking, dancing, or gardening over the past year); and (v) exhaustion (difficulty walking on uneven or inclined surfaces) [21].

#### 2.4.6. Disability

Functional status in basic (ADL) and instrumental (IADL) activities of daily living was evaluated using Subscale H of the MDS−HC instrument [15]. The assessment of ten basic ADLs—such as bed mobility, transfers, locomotion (both indoors and outdoors), dressing (upper and lower body), eating, toileting, personal hygiene, and bathing—covered the preceding three days. Each activity was scored on a scale ranging from 0 (fully independent) to 6 (complete dependence), with intermediate scores indicating varying degrees of assistance (e.g., setup help, supervision, limited or extensive support, or maximal assistance). For IADLs—including meal preparation, routine housekeeping, financial and medication management, telephone use, shopping, and transportation—participants’ ability over the past seven days was rated from 0 (independent) to 3 (entirely performed by others). Both ADL and IADL scores were consolidated into an 8-level hierarchical scale, where 0 reflected full independence and 7 denoted complete incapacity. Incident disability was defined as the onset of dependence in at least one of the following ADLs at the two-year follow-up: dressing, eating, toileting, bathing, bed mobility, locomotion, or transferring.

#### 2.4.7. Fall History and Fall Incidence

Fall history and fall incidence over the two-year period were assessed using item 5 of Section K in the MDS−HC [15], based on self-report or proxy report of any fall that occurred within 90 days prior to baseline or follow-up.

#### 2.4.8. Hospitalization

Time elapsed since the last hospital admission was evaluated using question 4 from section C of the MDS−HC [15].

#### 2.4.9. Mortality

Survival status was obtained from the participants’ general practitioners and was confirmed by the National Death Registry. Time to death was calculated from the date of the baseline visit to that of death. All events that occurred over 10 years from enrolment were included in the analysis.

### 2.5. Covariates

Height and weight were recorded using a standard stadiometer and a manual medical scale, respectively. Body mass index (BMI) was computed as weight in kilograms divided by the square of height in meters (kg/m^2^). Physical activity was assessed through participant self-report, based on their usual activity patterns during the 12 months preceding the interview [22]. Participants were asked to choose the category that best represented their behavior: (a) little to no activity; (b) mostly sedentary with occasional light walking or movement; (c) engagement in low-intensity activities (such as walking, dancing, fishing, or hunting) for 2–4 h weekly; (d) participation in moderate-intensity exercises (like running, uphill walking, swimming, or gymnastics) for 1–2 h weekly, or low-intensity activities for more than 4 h weekly; (e) moderate-intensity exercise exceeding 3 h per week; (f) frequent high-intensity exercise; or (g) walking more than 5 km on at least five days each week. Prior to data collection, participants were provided with clear explanations of what constituted low-, moderate-, and high-intensity physical activity. Smoking status was classified as current if the individual had regularly used tobacco at least once per week over the previous year [22]. The presence of multimorbidity was defined as having two or more of the following conditions: obesity, coronary artery disease, stroke, heart failure, peripheral vascular disease, hypertension, chronic respiratory diseases (including COPD, emphysema, or asthma), osteoarthritis, diabetes, dementia, Parkinson’s disease, kidney failure, or cancer [23]. Use of five or more medications concurrently was used to define polypharmacy [24,25].

### 2.6. Statistical Analysis

Continuous variables are presented as means ± standard deviations (SDs), while categorical and ordinal variables are reported as absolute numbers and percentages. Independent *t*-tests were used to assess differences in continuous variables according to the presence of dysphagia. Linear and multiple linear regression analyses were performed to examine the associations between dysphagia and physical performance measures, including IHG, WS, and 5STS. Binary logistic regressions were conducted to evaluate the associations between dysphagia and malnutrition, sarcopenia, frailty, ADL and IADL disability, history of falls, incident falls, and hospitalization. Ordinal logistic regressions were used to assess the relationship between dysphagia and cognitive function, as well as SPPB. Cox proportional hazards models were applied to identify predictors of survival, with time to death as the time variable. Dysphagia was treated as an independent and binary variable (presence vs. absence) in all analyses, while outcome measures were treated as dependent variables. All models were adjusted for age, sex, BMI, physical activity levels in the last year, smoking habits, multimorbidity, and polypharmacy. A significance level of 5% (*p* < 0.05) was adopted for all statistical tests, which were two-tailed. Statistical analyses were conducted using SPSS software (version 23.0; SPSS Inc., Chicago, IL, USA).

## 3. Results

### 3.1. Main Characteristics of Study Participants

The main characteristics of the study participants according to the presence of dysphagia are shown in Table 1. Older adults with dysphagia were older, had lower BMI and ASM values, and exhibited poorer performance on IHG and SPPB (overall and according to each domain). Furthermore, significant differences in the prevalence of sarcopenia, frailty, and malnutrition were observed between groups.

### 3.2. Cross-Sectional Associations Between Dysphagia and Health Parameters

Cross-sectional associations between dysphagia and health parameters are shown in Table 2. In the unadjusted analysis, dysphagia was significantly associated with IHG, WS, SPPB, sarcopenia, malnutrition, cognitive function, frailty, and ADL disability. After adjusting the analysis for covariates, only associations with IHG, WS, SPPB, and cognitive function remained significant.

### 3.3. Longitudinal Associations Between Dysphagia and Health Parameters

Longitudinal associations between dysphagia and adverse events are shown in Table 3. The results of the unadjusted analysis indicated that dysphagia was significantly associated with falls and death, but not with disability. However, these results were no longer significant after adjustment for covariates.

## 4. Discussion

The present study examined cross-sectional and longitudinal associations between dysphagia and various health parameters in a cohort of very old adults living in the community. Our findings indicate that dysphagia is cross-sectionally and significantly associated with poor physical performance and cognitive function. Although significance was found in the unadjusted models, dysphagia was no longer significantly associated with incident falls and death after adjustment for covariates.

To the best of our knowledge, this is the first investigation that examined the associations between dysphagia and a comprehensive set of health parameters. We found that dysphagia was significantly associated with low physical performance, but not with sarcopenia or frailty. On the other hand, many investigations have found significant associations between dysphagia and both sarcopenia and frailty in older adults [4,6,13,26,27,28,29].

A possible explanation for these divergent findings is most participants of the present study were likely in the early stages of dysphagia and not yet experiencing severe swallowing difficulties, which leads to reductions in caloric and protein intake, thereby causing malnutrition and contributing to structural and functional changes in the neuromuscular apparatus [6,7]. This view is in line with the low prevalence of dysphagia found in the present study (6.4%), the lack of significant associations with malnutrition, and the fact that participants were community-dwellings and mostly lived independently.

A complementary observation is that most investigations examining sarcopenia were conducted in hospitalized patients or individuals with specific characteristics (e.g., nursing-home residents) [28]. Furthermore, some studies have found that the development of frailty is dependent on the combination of both sarcopenia and dysphagia [30].

The initial presentation of dysphagia might also explain the lack of significant associations with important adverse events, including falls, disability, hospitalization, and death. Indeed, the major complications associated with dysphagia involve problems with efficacy and safety [1]. The first refers to an insufficient oral intake of nutrients, causing malnutrition, sarcopenia, and frailty, while the last embraces the risk of aspiration pneumonia, requiring hospitalization [1].

Both sarcopenia and frailty are well-established risk factors for numerous negative events, including falls, disability, hospitalization, and death [31,32,33,34,35]. Furthermore, some evidence has indicated that frailty has a crucial mediator role in the transition from muscle weakness to the outcomes examined in the present study [36]. In turn, the fact that dysphagia was operationalized according to the capacity to ingest specific foods only might suggest that participants of the present study had adapted to their conditions, reducing the risk of aspiration problems and deglutition.

The associations between dysphagia and cognitive function have long been acknowledged. Yatabe et al. [37] found that for every one-point increase in the Mini Mental State Examination, the risk of dysphagia was reduced by 14% in nursing home residents. Makhnevich et al. [38] noted that dysphagia and dementia often coexist in hospitalized patients, significantly increasing the risk of adverse events (e.g., respiratory complications). The results of a recent meta-analysis [39] are in line with these data, indicating significant associations between cognitive function and dysphagia in older adults with different chronic conditions (e.g., stroke, congestive heart failure).

The swallowing process is a highly complex sensory motor task that involves many brain areas, including prefrontal cortex, thalamus, and cingulate gyrus, among others [9,10]. These brain areas are also commonly activated during cognitive tasks with important impacts on the individuals’ capacity to perform daily activities, mainly those related to attention and inhibition [10]. The activation and efficiency of the neural networks underlying both sensorimotor and cognitive functions decline significantly with aging [6], which suggests that the associations observed in the present study may be due to the common and simultaneous effects of aging on the neural regions responsible for both functions.

The results of the present study have practical applications. The significant associations between dysphagia and both poor physical performance and reduced cognitive function emphasize the importance of comprehensive geriatric assessment. Holistic treatment strategies embracing multidomain interventions (e.g., physical exercise, cognitive training, deglutition maneuvers) need to be proposed to individuals with these characteristics.

The present study has limitations that should be acknowledged to enhance the interpretation of our results. First, the diagnosis of dysphagia was not confirmed through clinical or imaging examination. Second, sarcopenia was operationalized using an estimation of muscle mass by calf circumference rather than by using direct assessment tools. Third, longer follow-up periods might be necessary to capture associations between dysphagia and adverse outcomes. Fourth, malnutrition was assessed using a modified version of the GLIM criteria. Fifth, covariates, including physical activity levels, were assessed using self-reported measures. Finally, we examined a cohort of relatively healthy, very old adults living in a mountainous region. Therefore, extrapolations to other populations and settings should be made with caution.

## 5. Conclusions

The findings of the present study indicate that early-stage dysphagia is linked to deficits in physical and cognitive domains, underscoring the value of comprehensive geriatric assessment and the development of multidomain intervention strategies to properly manage the condition.

## Figures and Tables

**Table 1:** Characteristics of study participants according to the presence of dysphagia (*n* = 362).

Variable	Non-Dysphagia (*n* = 338)	Dysphagia (*n* = 24)	*p*-Value
Age, years	85.67 ± 4.81	88.77 ± 5.00	0.004
Body mass index, kg/m^2^	25.86 ± 4.41	21.83 ± 4.73	<0.001
Sex, women	65.9	81.8	0.124
SPPB, score	6.65 ± 3.90	1.09 ± 2.72	<0.001
Balance, score	2.62 ± 1.80	0.32 ± 1.04	<0.001
Walking speed, m/s	0.51 ± 0.29	0.11 ± 0.25	<0.001
5STS, s	15.21 ± 7.04	14.40 ± 1.85	<0.001
ASM, kg	15.33 ± 5.22	10.85 ± 5.14	<0.001
Handgrip strength, kg	31.48 ± 14.23	11.30 ± 12.34	<0.001
Sarcopenia, %	13.5	54.5	0.001
Frailty, %	67.8	94.7	0.014
Multimorbidity, %	22.1	13.6	0.352
Malnutrition, %	19.7	68.4	0.001
Polypharmacy, %	26.2	31.8	0.651
History of falls, %	13.6	14.3	0.930

Values are presented as mean ± standard deviation and prevalence (%). SPPB = short physical performance battery; ASM = appendicular skeletal muscle mass; 5STS = five times sit-to-stand test.

**Table 2:** Cross-sectional associations between dysphagia and health parameters.

Unadjusted				Adjusted		
Outcome	*Β*	*p*-value	95% CI (Lower, Upper)	*β*	*p*-value	95% CI (Lower, Upper)
5STS	2.887	0.920	−53.340, 59.123	30.279	0.329	−30.657, 91.215
IHG	**−20.1**	**0.001**	**−26.579, −13.782**	**−8.173**	**0.002**	**−13.434, −2.911**
4-MWS	**−0.396**	**0.001**	**−0.522, −0.269**	**−0.141**	**0.006**	**−0.241, −0.040**
SPPB	**3.407**	**0.001**	**2,382, 4,432**	**−2.542**	**0.001**	**−3.709, −1.375**
Cognitive function	**−2.562**	**0.001**	**−3.373, −1.750**	**1.051**	**0.022**	**0.153, 1.950**
Outcome	OR	*p*-value	95% CI (Lower, Upper)	OR	*p*-value	95% CI (Lower, Upper)
Sarcopenia	7.670	0.001	3.134, 18.768	0.552	0.295	0.182, 1.679
Frailty	8.550	0.039	1.114, 65.637	—	—	—
Malnutrition	8.841	0.001	3.232, 24.188	0.457	0.212	0.134, 1.561
ADL disability	16.574	0.001	4.793, 57.311	0.238	0.110	0.041, 1.386
IADL disability	—	—	—	—	—	—
History of falls	1.058	0.930	0.380, 3.734	1.178	0.818	0.293, 4.744
Hospitalization	1.196	0.780	0.342, 4.182	2.416	0.303	0.450, 12.955

**Bold denotes significance.** 5STS = five times sit-to-stand test; 4MWS = 4-min walking speed; ADLs = basic activities of daily living; IADLs = instrumental activities of daily living; CI = confidence interval; OR = odds ratio; SPPB = short physical performance battery; 5STS = five times sit-to-stand test. Adjusted for age, sex, body mass index, physical activity levels in the last year, smoking habits, multimorbidity, and polypharmacy.

**Table 3:** Longitudinal associations between dysphagia and health parameters.

Unadjusted				Adjusted		
Outcome	OR	*p*-value	95% CI (Lower, Upper)	OR	*p*-value	95% CI (Lower, Upper)
Falls	**4.604**	**0.023**	**1.23, 17.2**	0.349	0.154	0.08, 1.48
Disability	0.732	0.695	0.154, 3.483	0.860	0.313	0.444, 12.600
Outcome	HR	*p*-value	95% CI (Lower, Upper)	HR	*p*-value	95% CI (Lower, Upper)
Death	**0.627**	**0.034**	**0.407, 0.966**	0.810	0.376	0.508, 1.292

**Bold denotes significance.** CI = confidence interval; HR = hazard ratio; OR = odds ratio. Adjusted for age, sex, body mass index, physical activity levels in the last year, smoking habits, multimorbidity, and polypharmacy.

## Data Availability

Data are not publicly available due to privacy reasons, and can be obtained from Prof. Landi upon request.
